# Long‐term recurrence and brain metastasis of nasopharyngeal carcinoma mimicking cystic radiation encephalopathy relapse: a case report

**DOI:** 10.1186/s12883-021-02088-w

**Published:** 2021-02-08

**Authors:** Xuhui Chen, Lijie Ren, Guozhen Qiu, Liming Cao

**Affiliations:** 1 Department of Neurology, Peking University Shenzhen Hospital, 518000 Shenzhen, China; 2 Department of Neurology, Shenzhen University First Affiliated Hospital, 3002 Sungang West Road, Futian District 518000 Shenzhen, China; 3 Department of Neurology, Shenzhen Second People’s Hospital, 518000 Shenzhen, China; 4 Department of Neurology, The 3rd Affiliated Hospital of Shenzhen University, 518000 Shenzhen, China

**Keywords:** Nasopharyngeal carcinoma, Brain metastasis, Radiation encephalopathy, Magnetic resonance imaging, Case report

## Abstract

**Background:**

During medical imaging, cystic radiation encephalopathy and brain metastasis are difficult to differentiate, and hence they are easily misdiagnosed. To our knowledge, a nasopharyngeal carcinoma recurrence after more than seven years with cerebral metastasis that mimicked cystic radiation encephalopathy has not been reported.

**Case presentation:**

A 52-year-old man was admitted to the hospital owing to weakness of the right limb for one month, which increased in intensity for three days. He had been diagnosed with nasopharyngeal carcinoma in 2011, which was treated by radiotherapy. The patient successively developed cystic radiation encephalopathy and brain metastasis from the nasopharyngeal carcinoma, which mimicked cystic radiation encephalopathy relapse. Left frontotemporal craniotomy, surgical resection of brain metastasis, and repair of the skull base and dura were performed. Postoperative computed tomography showed that midline deviation recovered, and brain edema was reduced.

**Conclusions:**

This report is significant because brain metastasis from nasopharyngeal carcinoma can masquerade as a benign entity and cause fatal consequences. In patients presenting with cystic radiation encephalopathy, brain metastasis should be considered as a differential diagnosis.

## Background

Nasopharyngeal carcinoma (NPC) is a particularly prevalent carcinoma in Southern China for which radiotherapy is the primary treatment. A side effect is radiation encephalopathy (RE), a common and serious complication that is irreversible and life-threatening. The 3- and 5-year incidences of RE following radiotherapy are reported to be 10.8 and 34.9 %, respectively [1]. Another reason for treatment failure is recurrence or metastasis of NPC. Approximately 15–30 % of patients with NPC develop metastasis after completion of radiotherapy and chemotherapy [2]. The common sites for distant metastasis are the lungs, liver, bones, and retroperitoneal lymph nodes [3]. Brain metastasis of NPC is even rarer, ranging from 0.71 to 0.85 % of NPC cases [4, 5], and the clinical characteristics remain poorly understood. Differentiation between cystic RE and brain metastasis is difficult. A study [6] reported 14 % of the patients developed local recurrence, while 21 % had distant metastasis at the time of local relapse. Among those with NPC recurrence, 41 % had local recurrence detected within the first two years, 44 % in the 2nd to 5th year, and 15 % were detected > 5 years later. Most distant metastases in NPC occur within three years after radiotherapy completion [7].To our knowledge, an NPC recurrence after more than seven years with cerebral metastasis that mimicked cystic RE has not been reported. We present a patient with long-term recurrence and brain metastasis of NPC, which mimicked cystic RE, and a review of the literature. We provided useful information about the differential diagnosis between these two entities.

## Case presentation

In September 2017, a 52-year-old man was admitted to the hospital owing to weakness of the right limb for one month with increased intensity for three days. In 2011, he was treated for nasopharyngeal carcinoma with radiotherapy and chemotherapy. He had speech impairment, difficulty in mouth opening, dysphagia, and repeated pulmonary infections from the sequelae of NPC. Head computed tomography (CT) three years after onset indicated RE (film not available).

**Fig. 1:**
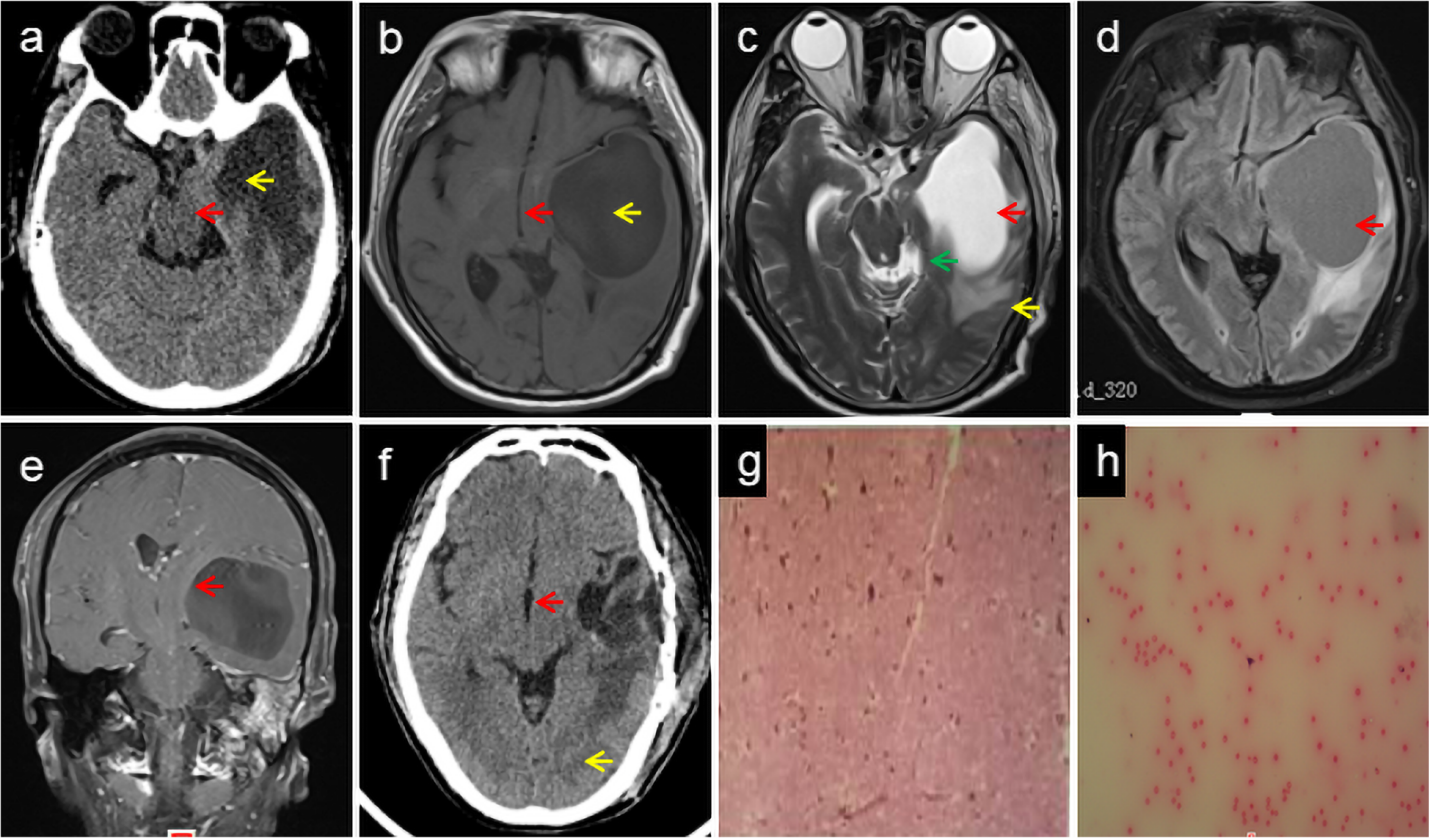
Neuroimaging and pathological findings in the first hospitalization. CT shows midbrain compression (**a**, red arrow) and herniation of falx cerebri caused by a massive solid-cystic lesion (**a**, yellow arrow). Axial T1-WI shows a massive cystic lesion in the left temporal lobe with short T1 signals (**b**, yellow arrow) and midline shift (**b**, red arrow). T2-WI shows abnormal long T2 signals (**c**, red arrow) in the cystic region with cerebral edema around the cyst (**c**, yellow arrow) and midbrain compression (**c**, green arrow). Flair imaging shows equal and even signals (**d**, arrow) in the cystic region. Coronal gadolinium-enhanced magnetic resonance imaging shows the cystic lesion is not enhanced (**e**, arrow) and did not extend from the skull base. Postoperative CT shows the midline shift (**f**, red arrow) and cerebral edema (**f**, yellow arrow) are alleviated. Pathological examination (**g**, hematoxylin & eosin stain, ×100) of the cystic wall of lesions and cyst-fluid smear examination (**h**) does not indicate the presence of tumor cells. CT: Computed tomography, Flair: Fluid attenuated inversion recovery, WI: weighted image

He had a history of anxiety and no history of major trauma, toxic exposure, or hereditary disease. Physical examination showed blood pressure of 90/61 mmHg, clear consciousness, vague speech, decreased memory, shallow nasolabial sulcus on the right, disappearance of pharyngeal reflex, decrease of muscle strength on the right upper and lower limbs (4/5), increased muscle tension in the right upper limb, and positive pyramidal sign on the right side. Emergency head CT showed solid-cystic lesions in the left temporal lobe and herniation of the cerebral falx (Fig. 1a). Laboratory analysis showed that his leukocyte count (10.94 × 10^9^/L) was elevated. C-reactive protein (30 mg/L), glycosylated hemoglobin (6.2 %), total cholesterol (7.78 mmol/L), triglycerides (1.93 mmol/L), and low-density lipoprotein cholesterol (4.93 mmol/L) levels were also higher. Fasting blood glucose, and liver and kidney function tests were normal. Electrocardiography showed T-wave changes. Brain magnetic resonance (MR) imaging (MRI) showed massive cystic lesions in the left temporal lobe and cerebral falx herniation (Fig. 1b-e). Brain MR angiography was normal. He was administered an antibiotic (cefoxitin), a diuretic (mannitol) to reduce intracranial pressure, and supportive treatment. On the fourth day after admission, he underwent partial excision of the cystic occupying lesion, internal decompression, and dural repair. On the sixth day after the operation, head CT (Fig. 1f) showed a midline shift, the cerebral edema was alleviated, and the right limb weakness was improved. Pathological examination (Fig. 1 g) of the cystic wall of lesions, as well as cyst-fluid smear examination (Fig. 1 h), detected no tumor cells.

**Fig. 2:**
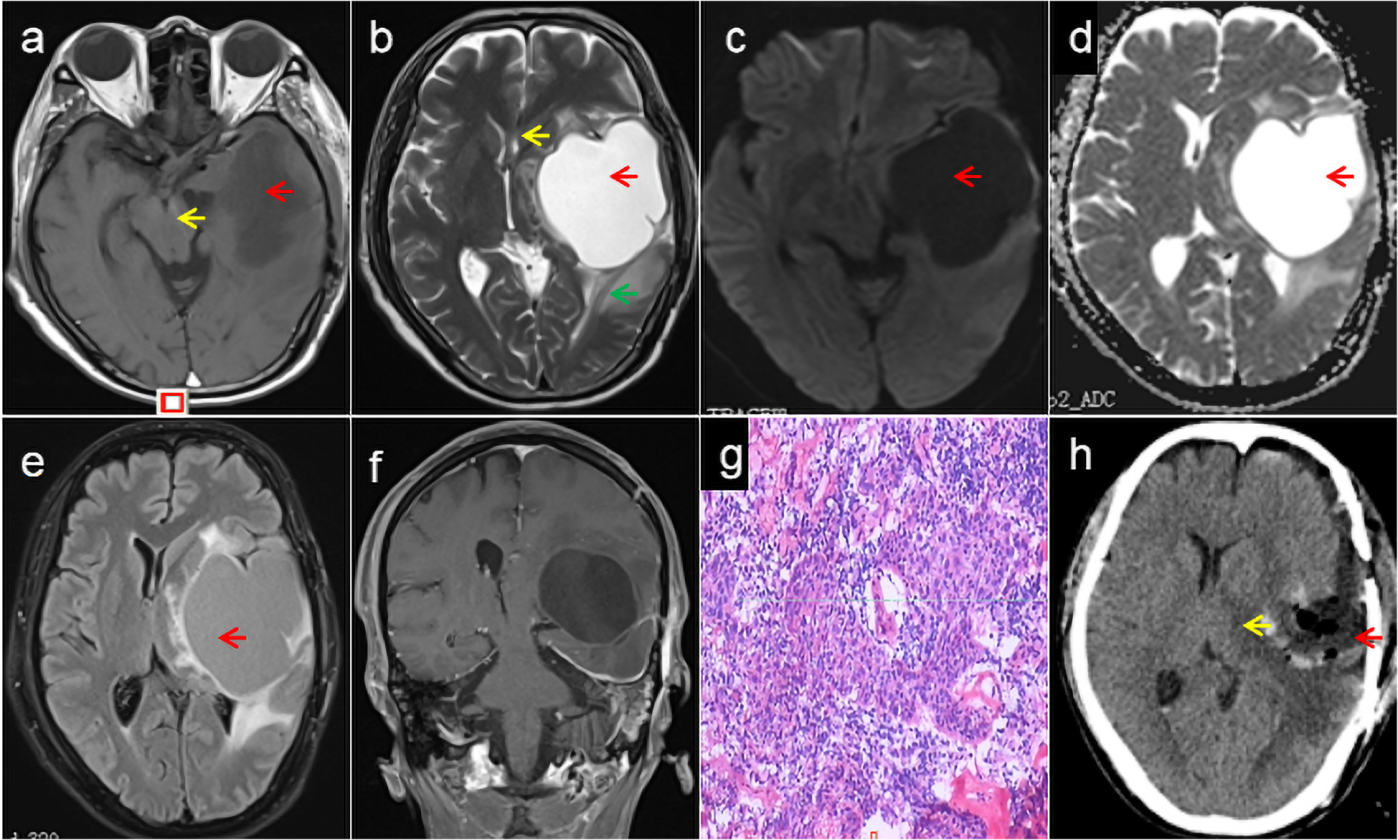
Neuroimaging and pathological findings in the second hospitalization. Axial T1-WI shows a massive cystic lesion (**a**, red arrow) in the left temporal lobe with short T1 signals, and midbrain compression (**a**, yellow arrow). T2-WI shows long T2 signals (**b**, red arrow) in the cystic region with cerebral edema around the cyst (green arrow) and midline shift (**b**, yellow arrow). The signal intensity of the cystic focus region is low on diffusion WI (**c**, arrow) and high on the apparent diffusion coefficient (**d**, arrow) map. Flair imaging shows equal and even signals in the cystic region (**e**) and enhanced magnetic resonance imaging shows no enhancement of the cystic lesion (**f**). Postoperative pathological examination of the cystic wall indicates infiltrative growth of heteromorphic epithelial cells and inflammatory cell infiltration (**g**, hematoxylin & eosin stain, ×150). Immunohistological examination shows p40 protein (+), p63 protein (+), cytokeratin (+), Ki67 protein (approximately 40%, +); These findings are consistent with those of the metastasis of differentiated non-keratinizing nasopharyngeal carcinoma. Postoperative computed tomography shows the reduced cyst (red arrow) and midline deviation (**h**, yellow arrow). Flair: Fluid attenuated inversion recovery, WI: weighted image

Six months later, he was readmitted owing to right limb weakness for one week in February 2018. Brain and nasopharynx MRI showed recurrence of cystic lesions in the left temporal lobe and cerebral hernia (Fig. 2a-f). Left frontotemporal craniotomy, surgical resection of brain metastasis, and repair of the skull base and dura were performed one week after admission. The pathology of the cystic wall indicated brain metastasis from NPC (Fig. 2 g). Postoperative CT showed that the cyst subsided, midline deviation recovered, and brain edema was reduced (Fig. 2 h). Follow-up chest CT demonstrated multiple metastases in both lungs one-and-a-half years postoperatively.

## Discussion and conclusions

In both evaluations of this patient (initial and recurrence), the cystic lesions did not extend from the skull base. Tumor cells were not found in the first pathological examination. Therefore, the first onset was diagnosed as cystic RE of the capsule wall. He had a history of RE as MRI imaging characteristics at the second onset were consistent with cystic RE; however, NPC metastasis was confirmed based on a combination of MRI imaging and the pathology of the capsule wall.

It is rare for NPC to metastasize to the brain through blood or cerebrospinal fluid circulation. A PubMed search revealed only a few well-described relevant cases that did not include spinal metastasis (Table 1 [5, 8–12]). Perhaps due to the effect of the blood-brain-barrier, the brain is not a typical site for metastasis of NPC [12]. Compared with all cases reported in Table 1, our case had the longest interval time from the diagnosis of NPC to the discovery of the brain metastasis. Some metastases in cases presented in Table 1 showed cystic changes with perifocal edema, although these cystic changes were not as large as in our case, with malignant edema mimicking cystic RE.

**Table 1:** Detailed report of brain metastasis from nasopharyngeal carcinoma

Author/ year	Sex/ age	Major treatment for NPC	Site in the brain	Systemic metastasis	Pathology of brain metastatic focus	EBV detection	Time interval between onset/ diagnosis of NPC and identification of brain metastasis	Perifocal edema of metastases	Morphology of brain metastases	Enhancement change	Treatment for brain metastases
Liaw et al./1994 [5]	Male/69	nd	Bilateral occipital	+ bone	Non-keratinizing squamous cell carcinoma	nd	2 years	+	Cystic and Non cystic	+	Chemoradiotherapy.
Ngan et al./2002 [8]	Male/33	Chemoradiotherapy	Left occipital	+ lung	Undifferentiated carcinoma	+	51 months	+	Cystic	nd	Surgery
Özyar et al./2004 [9]	Male/41	Radiotherapy	Right temporal lobe	-	Undifferentiated carcinoma	-	45 months	+	Non cystic	Homogenously enhancing	Whole brain radiotherapy
Kaidar-Person O et al/2012 [10]	Male/56	Chemoradiotherapy	Occipital	+ lung	Undifferentiated carcinoma	+	3 months	nd	nd	nd	Radiotherapy
Kuo CL et al./ 2014 [11]	female/37	Chemoradiotherapy	Dura and frontotemporal lobe	-	Differentiated non-keratinizing carcinoma	+	7 months	+	Non cystic	Heterogeneously enhancing	Chemoradiotherapy and surgery
Su Z et al/2019 [12]	Male/43	Chemoradiotherapy	Brain and frontal bone	-	Undifferentiated non-keratinizing carcinoma	-	3.5 years	Not obvious	Non cystic	+	Chemoradiotherapy

+ Positive or detected, - Negative or not detected; *nd *Not determined, *EBV* Epstein-Barr virus, *NPC *Nasopharyngeal carcinoma

At first, the pathophysiology of RE involves brain tissue edema and vascular endothelial injury after radiotherapy, subsequent brain tissue necrosis, gliosis, and granulation tissue formation, followed by encephalomalacia of the lesions and cystic necrosis [13]. The cyst is formed by liquefaction and brain tissue necrosis, and as the fluid and capacity of the cyst increase, it compresses the surrounding brain tissue and causes brain herniation. In our case, cystic RE showed a low signal in T1 weighted MRI images (WI), a high signal in T2 WI, and well-defined round or oval lesions of hyperintensity on T2 WI [14], similar to the characteristics of cystic brain metastasis. Metastasis is a promoter of cystic changes, as tumor cells secrete sufficient amounts of proteins to promote cyst growth, and cancer cells can aggravate vasogenic edema. MRI is a useful and noninvasive method to evaluate RE and its evolution but is usually focused on the temporal lobe. Solid enhanced nodular foci were the earliest MRI abnormalities observed in the patient’s RE [14]. Brain metastatic foci can be abnormally enhanced in severe edema with a short T1 WI signal [5, 8]. It is difficult to distinguish RE and metastasis by conventional MR sequences, but this can be achieved by perfusion-weighted imaging.

The quantitative detection of serum Epstein-Barr virus (EBV) deoxyribonucleic acid (DNA) may be an indicator for screening and monitoring the recurrence of NPC, and patients with elevated plasma EBV DNA levels are more likely to relapse [15]. However, most of the EBV test results in the cases in Table 1 were negative. The majority of NPCs consist of poorly differentiated squamous cell carcinoma with high degrees of malignancy, and they are highly sensitive to radiotherapy. Individual radiosensitivity is determined by the proportion of lethal chromosomal aberrations *in vitro* irradiated lymphocytes that might have the potential to predict the morbidity and severity of RE, specifically, patients with a high radiosensitivity had shorter latency of RE than those with low or intermediate radiosensitivity [16]. Some RE patients can obtain symptomatic relief with high dose corticosteroids in the early stage; if lesions are metastatic, the symptoms may rebound. Operations to reduce intracranial pressure and inflammatory responses can be performed in cystic RE patients with severe symptoms and signs [17]. Our patient was satisfied with the treatments he received and his recovery.

This unique case is notable owing to the extended period before recurrence and brain metastasis from NPC mimicking cystic RE. Hence, multicenter and large-sample clinical studies are needed to confirm the characteristics of brain metastasis from NPC. In conclusion, we determined that when patients present with cystic RE, brain metastasis should be considered as a differential diagnosis.

## Data Availability

Data sharing not applicable to this article as no datasets were generated or analyzed during the current study.

## Abbreviations

NPC: Nasopharyngeal carcinoma
RE: Radiation encephalopathy
CT: Computed tomography
MR: Magnetic resonance
MRI: Magnetic resonance imaging
WI: Weighted image
